# Fate of Residual Aorta After Surgery for Type A Aortic Dissection

**DOI:** 10.21470/1678-9741-2024-0243

**Published:** 2025-04-28

**Authors:** Apeksha Mittal, Pankaj Aggarwal, Harkant Singh, Manphool Singhal, Arun Sharma, M. R. Mohamed Irshad, Nishit Santoki, Nitish Kumar, Dollphy Garg, Chandra Shekhar Singh Aswal, Richa Soni, A. Arun George

**Affiliations:** 1 Cardiothoracic Vascular Surgery, Post Graduate Institute of Medical Education and Research, Chandigarh, Chandigarh, India; 2 Department of Anesthesia, Post Graduate Institute of Medical Education and Research, Chandigarh, Chandigarh, India; 3 Radiodiagnosis and Imaging, Post Graduate Institute of Medical Education and Research, Chandigarh, Chandigarh, India

**Keywords:** Abdominal Aorta, Thoracic Aorta, Aortic Dissection, Aneurysm, Pathologic Dilatation, Thrombosis, Tomography

## Abstract

**Introduction:**

Surgical treatment of type A aortic dissection is essentially palliative. Many patients who undergo the procedure still have a dissection flap in the residual aorta, with a persistent patent or partially thrombosed false lumen leaving them susceptible to the dilatation of distal aorta and aneurysm formation.

**Methods:**

Patients who had undergone surgery for type A aortic dissection from January 2015 till December 2022 were recruited into the study. Two follow-up computed tomography scans were performed at least six months apart, the first one at least one month after the surgery.

**Results:**

A persistent dissection flap was found in 34 (68%) patients. All segments of residual distal aorta showed dilatation with time. Growth rate was maximum for abdominal aorta - 3.1 (1.6 - 5.4) mm/year. Patency of false lumen was the only significant factor associated with growth of lower descending thoracic aorta and abdominal aorta (P<0.05). Maximum growth was seen in the patients with partial thrombosis of the false lumen, followed by those with patent false lumen. Two patients with partially thrombosed false lumens required reintervention in the form of endovascular stenting.

**Conclusion:**

Patients after surgery for type A aortic dissection with partially thrombosed false lumens are more prone to aortic dilatation. Regular follow-up of these patients with computed tomography aortogram can lead to timely detection of these sequalae and intervention as needed.

## INTRODUCTION

**Table:** 

Abbreviations, Acronyms & Symbols	
ACP	= Antegrade cerebral perfusion
BMI	= Body mass index
CCA	= Common carotid artery
CKD	= Chronic kidney disease
CLD	= Chronic liver disease
CT	= Computed tomography
DHCA	= Deep hypothermic circulatory arrest
DTA	= Descending thoracic aorta
FL	= False lumen
IQR	= Interquartile range
RA-IVC	= Right atrial and inferior vena cava
SD	= Standard deviation
TAAD	= Type A aortic dissection

Acute type A aortic dissection (TAAD) remains one of the most challenging conditions for cardiovascular surgeons. Patients with acute TAAD who do not receive treatment have mortality at a rate of 1-2% per hour during the first day, and almost half die by one week. Death is caused by proximal or distal extension of dissection, valvular dysfunction, pericardial tamponade, arch vessel occlusion causing stroke, visceral ischaemia, or rupture resulting in a mortality of about 20% on day one and 30% in 48 hours^[1]^. The crucial elements for surgical success in acute TAAD are the excision of the primary entry tear, correction of any aortic valve insufficiency, and in order to correct distal malperfusion, restoration of dominant true lumen flow in the downstream aorta^[2]^.

However, surgical treatment remains essentially palliative, as most operative survivors have a residual dissected aorta, often with a patent false lumen (FL). This exposes patients to distal aortic dilatation and subsequent aneurysm formation, with its inherent risks of aortic rupture or reoperation^[3]^.

So, in the present study, we assessed the growth rate of residual distal aorta, risk factors for aortic dilatation after surgery, and need for reintervention.

## METHODS

The study was carried out after approval of the Institution’s Ethics Committee (IEC-INT/2022/MCh-275). Patients who had undergone surgery for TAAD from January 2015 till December 2022 were recruited into the study after signed the informed consent. Patients with preexisting kidney disease were excluded from the study, so as to avoid contrast injection during computed tomography (CT) scan.

Baseline patient information like demographics, risk factors, preoperative condition, CT and/or transesophageal echocardiographic findings, intraoperative notes, and postoperative outcomes were reviewed and noted from the available clinical records. Two follow-up CT scans were performed at least six months apart, the first one at least one month after the surgery. The true and false lumen diameters were measured at distal aortic arch just distal to the origin of left common carotid, upper descending thoracic aorta (DTA) at the level of bifurcation of the pulmonary arteries, lower DTA at the level of diaphragm, and abdominal aorta at the origin of the renal artery. All the scans were also studied for the status of the FL, *i.e.*, completely patent (blood flow with no thrombus seen), partially thrombosed (both blood flow and thrombus seen), and completely thrombosed (only thrombus seen and no flow).

### Statistical Analysis

Normality of data was assessed by Kolmogorov-Smirnov test. Normally distributed quantitative variables were expressed using mean and standard deviation. Skewed variables were expressed as median and interquartile range. For normally distributed data, mean was compared using independent *t*-test. For non-normally distributed data, median was compared using Mann-Whitney U test and Wilcoxon signed rank test. Difference in more than two groups was compared by Kruskal-Wallis H test, followed by post hoc test. Qualitative data was expressed in numbers and percentage, and Chi-square test and Fisher’s exact test were used for comparison. Statistical difference between the proportions of paired data was tested by McNemar test. *P* < 0.05 was considered statistically significant. The statistical analysis was carried out using IBM Corp. Released 2011, IBM SPSS Statistics for Windows, version 20.0, Armonk, NY: IBM Corp.

## RESULTS

A total of 66 patients underwent surgery for TAAD, from January 2015 to December 2022, at the Post Graduate Institute of Medical Education and Research, Chandigarh, India. Out of these, 14 patients (21.2%) died after surgery. The causes of death included low cardiac output syndrome (n=5), sepsis (n =4), neurological complications (n=2), bleeding (n=2), and mesenteric ischemia (n=1). Out of the 52 patients who were discharged, two patients succumbed at home with unknown cause of death. So, a total of 50 patients were followed and recruited into the study. Mean follow-up period was 4.5 years. Patients’ characteristics were shown in Table 1.

**Table 1:** Patients’ characteristics.

Characteristics	Values
Age (years + SD)	44 + 13
Sex (male: female)	7:03
Anthropometry	
Height (cm + SD)	168.8 + 10.3
Weight (kg + SD)	69.5 + 24.4
BMI (kg/m^2^ + SD)	24.4 + 4.0
Comorbidities, n (%)	
Diabetic	9 (18%)
Hypertensive	27 (54%)
CKD	4 (8%)
Marfanoid	8 (16%)
Smoking	16 (32%)
Hypothyroid	4 (8%)
Prior aortic valve replacement	2 (4%)
Post renal transplant	1 (2%)
Pregnancy	1 (2%)
CLD	1 (2%)

BMI=body mass index; CKD=chronic kidney disease; CLD=chronic liver disease; SD=standard deviation

### Operative Details

The patients were planned for surgery as soon as possible after imaging. The details of surgery are summarized in Table 2.

**Table 2:** Operative details.

Operative details	Values
Proximal operation, n (%)	
Root replacement with valved conduit	33 (66%)
Mechanical	31 (62%)
Bioprosthetic	2 (4%)
Supracoronary ascending aorta replacement	13 (26%)
Aortic valve resuspension with ascending aorta replacement	3 (6%)
Valve-sparing aortic root replacement	1 (2%)
Distal operation, n (%)	
Hemiarch	38 (76%)
Total arch	9 (18%)
Isolated ascending aorta	2 (4%)
Total arch + elephant trunk	1 (2%)
Arterial cannulation, n (%)	
Right axillary	33 (66%)
Right axillary + femoral	4 (8%)
Right axillary + left CCA	3 (6%)
Left CCA	2 (4%)
Femoral	2 (4%)
Aortic	2 (4%)
Right CCA + femoral	1 (2%)
Right CCA	1 (2%)
Left CCA + femoral	1 (2%)
Right femoral + brachiocephalic artery	1 (2%)
Venous cannulation, n (%)	
RA-IVC dual stage	41 (82%)
Bicaval	8 (16%)
Femoral	1 (2%)
Temperature (°C + SD)	26.1 + 2.3
ACP, n (%)	45 (90%)
DHCA, n (%)	3 (6%)
Size of conduit (mm + SD)	24.1 + 2.5
ACP time (min), median (IQR)	49.5 (15)
DHCA time (min + SD)	27.7 + 2.5
Aortic cross-clamping time (min + SD)	207.7 + 58.7
Cardiopulmonary bypass time (min + SD)	296.3 + 58.5

ACP=antegrade cerebral perfusion; CCA=common carotid artery; DHCA=deep hypothermic circulatory arrest; IQR=interquartile range; RA-IVC=right atrial and inferior vena cava; SD=standard deviation

### Dimensions of Aortic Segments

**Fig. 1:**
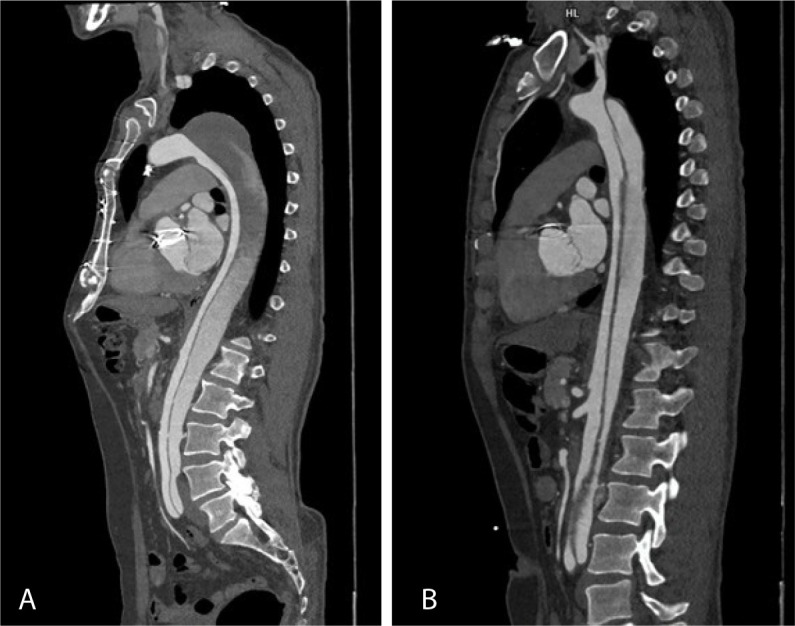
Sagittal section from postoperative computed tomography aortograms of two patients showing dissection flap in descending thoracic aorta with a) partially thrombosed false lumen and b) patent false lumen.

Two CT aortograms performed at least six months apart were analyzed. A persistent dissection flap was found in 34 (68%) patients. The mean dimensions of various aortic segments and their growth rates have been summarized in Table 3. Growth rate was maximum for abdominal aorta, 3.1 (1.6 - 5.4) mm/year. Two patients developed DTA aneurysm, diameter > 55 mm, and required endovascular stenting. Patency of FL was also studied in the two CT scans (Figure 1), and any change was noted (Table 4).

**Table 3:** Dimensions of aortic segments and their growth rates.

Aortic segments	Dimensions in the first CT (mm) Median (IQR)	Dimensions in the last CT (mm) Median (IQR)	Rate of growth (mm/year) Median (IQR)
Aortic arch	27.6 (24.3-30.3)	28.0 (25.6-32.3)	1.5 (0.1-3.9)
Upper DTA	28.9 (25.0-33.9)	32.2 (28.0-36.0)	2.8 (0.8-6.7)
Lower DTA	25.0 (20.7-30.0)	26.3 (22.1-32.9)	2.9 (1.2-6.0)
Abdominal aorta	24.0 (20.0-27.8)	26.0 (22.0-32.9)	3.1 (1.6-5.4)

CT=computed tomography; DTA=descending thoracic aorta; IQR=interquartile range

**Table 4:** Change in patency of false lumen.

	First CT	Last CT	*P*-value
Patent	19 (52%)	17 (46%)	0.82
Partial thrombosis	12 (32%)	12 (32%)	
Thrombosed	6 (16%)	8 (22%)	

CT=computed tomography

### Risk Factors Associated with Growth Rate of Different Aortic Segments

Various risk factors like hypertension, diabetes mellitus, connective tissue disorders, history of cigarette smoking, bicuspid aortic valve, distal extent of dissection flap, and FL patency were studied for their association with increase in diameter of different aortic segments, as shown in Table 5. Partial thrombosis of the FL was only significantly related to the growth of lower DTA and abdominal aorta.

**Table 5:** Association of different factors with rate of growth of different aortic segments.

	Aortic arch (*P*-value)	Upper DTA (*P*-value)	Lower DTA (*P*-value)	Abdominal aorta (*P*-value)
Diabetes mellitus	0.68	0.06	0.21	0.88
Hypertension	0.57	0.28	0.1	0.25
Connective tissue disorder	0.87	0.91	0.28	0.18
Smoker	0.5	0.95	0.12	0.8
Aortic valve morphology	1	0.6	0.75	0.56
Distal extent	0.58	0.69	0.16	0.08
False lumen patency	0.78	0.44	0.04	0.01

DTA=descending thoracic aorta

## DISCUSSION

Ascending aortic dissection is a relatively less common and challenging pathology affecting on an average three to four individuals per 100,000 people per year, with very high mortality, if there is a delay in the diagnosis or the treatment^[4]^. Although the immediate surgical outcomes of acute TAAD have recently improved with advances in surgical techniques and perioperative care, the long-term fate of residual FL of the distal aorta after repair is still being studied.

### Change in Patency of False Lumen with Time

Despite repair, FL tends to remain patent in a significant number of patients, as seen from postoperative CT. No dissection flap was found in 16 (32%) patients. Out of 34 patients with persistent dissection flap, patent FL was found in 19 (52%) patients in the first imaging compared to 17 (46%) patients in the last one. Partially thrombosed FLs were found in 12 (32%) patients in both CTs. Thrombosed FL was found in six (16%) patients in the first CT compared to eight (22%) patients in the last CT. However, the difference was not statistically significant (*P* = 0.82).

Similar studies from other centres also show that most of the patients in the postoperative period tend to have a persistent patent FL in the residual aorta^[5^-^9]^. The distal FL remains patent when it is persistently being perfused. It can be either due to additional entry tears that have not been resected during the surgery or due to formation of new entry tears, that can form while placing an aortic cross-clamp on the already friable dissected aorta^[10]^.

### Growth Rate of Distal Aorta

All segments of residual distal aorta showed dilatation during follow-up. Growth rate was maximum for abdominal aorta - 3.1 (1.6 - 5.4) mm/year.

Various risk factors like hypertension, diabetes, connective tissue disorders, history of cigarette smoking, bicuspid aortic valve, distal extent of dissection flap, and FL patency were studied for their association with increase in diameter of different aortic segments. Partial thrombosis of the FL was only significantly related to the growth of lower DTA and abdominal aorta. No significant factor was found for aortic arch or proximal DTA. Two patients developed DTA aneurysm, diameter > 55 mm, and required endovascular stenting. Both patients had partially thrombosed FL.

Multiple studies have demonstrated that the status of FL patency is related to the rate of growth of the residual aorta. Other factors like hypertension and baseline diameter of the aorta, which have been significant predictors of growth in these studies, were not significant in our study^[11^-^13]^.

However there have been differences regarding whether partially thrombosed FL is more protective over completely patent FL, or whether it predicts faster growth rate, with the exact pathophysiology being largely unknown^[5^,^6^,^14]^. One possible explanation is that when the FL is fully patent, blood enters the FL via an entry tear and comes out via an exit/re-entry tear. However, in patients with partially thrombosed FL, the exit site may get blocked by the thrombus, thus leading to reduction in the outflow of blood from the FL. This can cause pressurization of the FL and increased tension in its already thinned out wall, which may contribute to subsequent aortic dilatation. Another potential mechanism is related to hypoxia in the aortic wall, adjacent to the thrombus. This reduced oxygen supply to the aortic wall in the region of the thrombus causes activation of inflammatory cascade and triggers neovascularization, which ultimately results in weakening of the aortic wall. This weakening could further contribute to aortic dilation and compromise the long-term structural integrity of the aorta^[10]^.

### Limitations

Our study has several limitations. The first being related to a relatively small sample size and short duration of follow-up. So, the results cannot be generalized and don’t accurately reflect the late outcomes after surgery. Secondly, patients got CT aortograms done at different intervals of time after surgery, thus affecting the uniformity. Further studies with a larger number of patients and longer follow-up period with a uniform protocol of getting CT aortograms are needed to enhance the existing knowledge.

## CONCLUSION

The present study revealed that there is dilatation of residual aorta after surgery for TAAD, which affects all parts of the aorta. Clearly, patients with partial thrombosis of FL had rapid growth of aorta and needed reintervention. Moreover, FLs don’t thrombose over time. So, we believe that systematic follow-up of the patients undergoing surgery for TAAD is necessary for early diagnosis and management of late complications.
